# ARMC5 selectively degrades SCAP-free SREBF1 and is essential for fatty acid desaturation in adipocytes

**DOI:** 10.1016/j.jbc.2024.107953

**Published:** 2024-11-02

**Authors:** Akifumi Uota, Yosuke Okuno, Atsunori Fukuhara, Shugo Sasaki, Sachiko Kobayashi, Iichiro Shimomura

**Affiliations:** 1 Department of Metabolic Medicine, Osaka University Graduate School of Medicine, Suita, Osaka, Japan; 2 Department of Adipose Management, Osaka University Graduate School of Medicine, Suita, Osaka, Japan

**Keywords:** ARMC5, SREBF1, adipocytes, fatty acid desaturation, ubiquitination

## Abstract

SREBF1 plays the central role in lipid metabolism. It has been known that full-length SREBF1 that did not associate with SCAP (SCAP-free SREBF1) is actively degraded, but its molecular mechanism and its biological meaning remain unclear. ARMC5–CUL3 complex was recently identified as E3 ubiquitin ligase of full-length SREBF. Although ARMC5 was involved in SREBF pathway in adrenocortical cells, the role of ARMC5 in adipocytes has not been investigated. In this study, adipocyte-specific *Armc5* KO mice were generated. In the white adipose tissue of these mice, all the stearoyl-CoA desaturase (*Scd*) were drastically downregulated. Consistently, unsaturated fatty acids were decreased and saturated fatty acids were increased. The protein amount of full-length SREBF1 was increased, but ATAC-Seq peaks at the SREBF1-binding sites were markedly diminished around the *Scd1* locus in the WAT of *Armc5* KO mice. Armc5-deficient 3T3-L1 adipocytes also exhibited downregulation of *Scd*. Mechanistically, disruption of *Armc5* restored decreased full-length SREBF1 in CHO cells deficient for *Scap*. Overexpression of *Scap* inhibited ARMC5-mediated degradation of full-length SREBF1, and overexpression of *Armc5* increased nuclear SREBF1/full-length SREBF1 ratio and SREBF1 transcriptional activity in the presence of exogenous SCAP. These results demonstrated that ARMC5 selectively removes SCAP-free SREBF1 and stimulates SCAP-mediated SREBF1 processing, hence is essential for fatty acid desaturation *in vivo*.

ARMC5 is a member of ARMC subfamily and consists of two protein-interaction domains, Armadillo repeats and BTB domain (1). In 2013, *ARMC5* gained particular attention because somatic loss-of-function mutation in adrenocortical cells causes primary bilateral macronodular adrenal hyperplasia, which produce excess cortisol and develop Cushing syndrome (2). However, the molecular function of ARMC5 had been obscure until recently.

In 2020, Cavalcante *et al.* found that ARMC5 ubiquitinated and degraded itself through interaction with CUL3, a part of E3 ubiquitin ligase, by the BTB domain (3). This finding raised the possibility that ARMC5–CUL3 complex may degrade not only ARMC5 itself but also yet-unknown protein which would interact with the Armadillo repeats of ARMC5 (3). Accordingly, we (4) and others (5, 6) identified the potential targets of ARMC5–CUL3 complex through different approaches. One group employed yeast two-hybrid assay (7) and affinity-capture mass spectrometry (MS) (5) using ARMC5 as bait and found that ARMC5 was a part of an RPB1 (the largest subunit of RNA polymerase II (Pol II))-specific ubiquitin ligase. Loss of ARMC5 increased RPB1 protein and enlarged Pol II pool, although the significance of enlarged Pol II pool remained unclear (5, 8). Another group examined gene expressions in the adrenocortical cells introduced with siRNA targeting *Armc5* and found that ARMC5 might be involved in NRF1 ubiquitination and regulated p38 pathway, ferroptosis, and redox homeostasis (6).

We identified the interaction of ARMC5 and SREBF through affinity-capture MS in the 3T3-L1 adipocytes using SREBF1 as bait (4). SREBF is a transcription factor which participates in fatty acid synthesis and cholesterol metabolism; SREBF1 mainly regulates lipogenesis and SREBF2 mainly regulates cholesterol metabolism. Newly synthesized SREBF is inserted into the endoplasmic reticulum (ER) as full-length SREBF and forms complex with SREBF chaperone (SCAP). SCAP is a membrane protein consisting of eight transmembrane (TM) helicases and WD40 repeat motifs. TM helices 2-6 comprise the sterol-sensing domain and WD40 repeats mediate binding to SREBF. In sterol-depleted cells, the SCAP binds to COPII coat proteins and SCAP/SREBF complex move from ER to the Golgi apparatus (Golgi), where the N-terminus of SREBF is released by two sequential cleavages. An initial cleavage is mediated by MBTPS1 near the middle of the lumenal loop and the second cleavage is mediated by MBTPS2 at a site within the first TM segment (9). Released N-terminus of SREBF translocates to the nucleus and works as a transcription factor (designated as nuclear SREBF) (10) (Fig. 1*A*). When cholesterol builds up in the ER membranes, the sterol binds to SCAP and induces a conformational change of SCAP. Then, SCAP binds to INSIG and can no longer bind to COPII coat proteins (11). Importantly, as ARMC5 is localized to the cytosol, ARMC5–CUL3 complex degrades full-length SREBF on the ER, but not nuclear SREBF in the nucleus (4).

**Figure 1:**
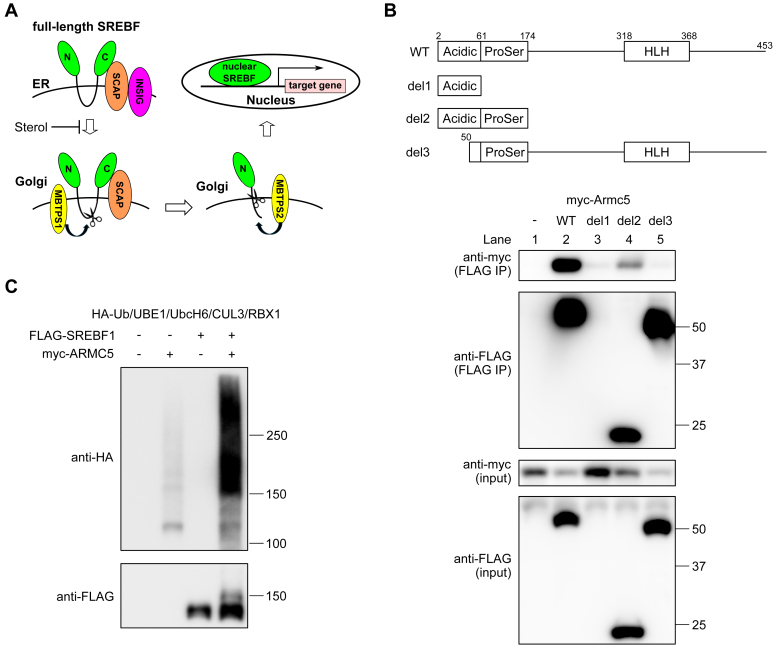
**Expanded analysis of ARMC5-mediated ubiquitination of full-length SREBF1 *in vitro*.***A*, a schematic of the processing of SREBF1. Full-length SREBF is inserted into the ER and forms complex with SCAP and INSIG. In sterol-depleted cells, INSIG dissociates from SCAP and SREBF/SCAP complex moves to the Golgi, where N terminus of SREBF is released by two sequential cleavages by MBTPS1 and MBTPS2. Released SREBF translocates to the nucleus and works as transcription factor. *B*, Western blotting of lysates (input) or samples immunoprecipitated with anti-FLAG antibody (FLAG IP) from the HEK293T cells transfected with pcDNA3.1-myc-mArmc5, pcDNA3.1-FLAG (−), pcDNA3.1-FLAG-Srebf1(N) (WT), or indicated mutant of pcDNA3.1-FLAG-Srebf1(N) (del1-3) with the indicated antibodies. *C*, immunoprecipitated FLAG-SREBF and/or purified myc-ARMC5 were added to the *in vitro* ubiquitination assay in the presence of HA-Ub, UBE1, UbcH6, and CUL3/RBX1. The reaction products were blotted with indicated antibodies. acidic, acidic domain; ProSer, serine/proline-rich domain; HLH, basic helix-loop-helix leucine zipper region.

Although *Armc5* and *Srebf* were ubiquitously expressed, the roles of *Armc5* in various tissues other than adrenal cortex were scarcely known. Whole body *Armc5* KO mice died during early embryonic development in the pure strain (7, 12). *Armc5* KO mice could survive with smaller body size and compromised T-cell immune responses in the C57BL/6J x 129/sv F1 background (7) or neural tube defect in the C57BL/6 x CD1 background (8). As SREBF1 is the master regulator of fatty acid synthesis and the adipose tissue is one of the principal sites in fatty acid metabolism, ARMC5 might play important roles in the adipocytes. To examine our hypothesis *in vivo*, we set out the generation of the mice where *Armc5* was disrupted specifically in the adipocytes.

## Results

### Expanded analysis of ARMC5-mediated ubiquitination of full-length SREBF1 *in vitro*

We previously reported that ARMC5 physically interacted with and ubiquitinated full-length SREBF1 in a CUL3-dependent manner. Before proceeding to *in vivo* experiments, we expanded these previous findings *in vitro*. In the previous study, we reported the interaction between Armadillo repeats of ARMC5 and N-terminus of SREBF1 (mouse SREBF1a, 2–453 a.a.), which consists of transactivation acidic domain, serine/proline-rich domain, and basic helix-loop-helix leucine zipper (bHLH-LZ) region (4) (Fig. 1*B*). To narrow down the ARMC5-interacting region of SREBF1, we transfected *Armc5* and serial deletion mutants of *Srebf1* into HEK293T cells and performed co-immunoprecipitation. Acidic domain and serine/proline-rich domain of SREBF1 was sufficient for interaction with ARMC5 (lane 4 *versus* 1, Fig. 1*B*) and acidic domain was necessary for interaction with ARMC5 (lane 5 *versus* 2, Fig. 1*B*). Unfortunately, acidic domain alone was not expressed in HEK293T cells (lane 3, Fig. 1*B*). These data indicated that ARMC5–SREBF1 interaction was mediated between acidic domain/serine-proline rich domain of SREBF1 and Armadillo repeats of ARMC5. Next, we performed *in vitro* ubiquitination assay to confirm ARMC5-mediated ubiquitination of full-length SREBF1 (4). As ARMC5-mediated ubiquitination of RPB1 was recently demonstrated through *in vitro* ubiquitination assay (5), we employed the similar protocol. When FLAG full-length SREBF1 and myc-ARMC5 were incubated with UBE1, UbcH6, CUL3, RBX1, and HA-Ubiquitin, ubiquitinated protein more than 150 kDa (Fig. 1C, upper panel) and a 150 kDa ubiquitination band of FLAG-SREBF1 (Fig. 1, C, lower panel) were visible, demonstrating that ARMC5 is a *bona fide* ubiquitin ligase for full-length SREBF1.

### Generation of adipocyte-specific *Armc5* KO mice (AdArmc5 KO)

**Figure 2:**
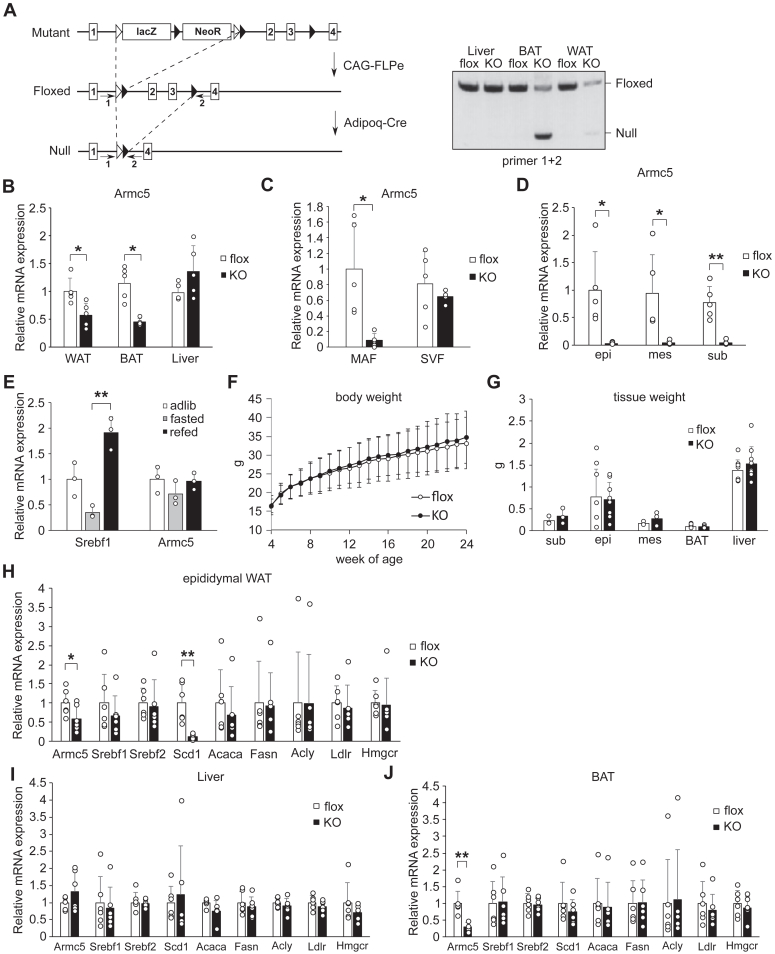
**Generation of AdArmc5 KO and downregulation of Scd1 in the WAT of AdArmc5KO.***A*, a strategic scheme (*left*) and genotyping (*right*) for target disruption of mouse Armc5. The number in the square represents the number of exon of mouse Armc5. Arrows with number represent the primers used in genotyping. *B*, gene expression of Armc5 in the indicated tissues of 15-week-old Armc5 flox (flox) or AdArmc5 KO (KO) (n = 5, each). All the values were expressed relative to the WAT of Armc5 flox. *C*, gene expression of Armc5 in the MAF and the SVF prepared from the epididymal WAT of 15-week-old Armc5 flox (flox) or AdArmc5 KO (KO) (n = 5, each). All the values were expressed relative to the MAF of Armc5 flox. The primers used in RT-qPCR recognized the exon 3 of Armc5. *D*, gene expression of Armc5 in the MAF prepared from epididymal WAT (epi), mesenteric WAT (mes), and subcutaneous WAT (sub) of 28-week-old Armc5 flox (flox) or AdArmc5 KO (KO) (n = 5, each). *E*, gene expression of the indicated genes in the epididymal WAT of 10-week-old C57BL/6J fed ad libitum (adlib) or fasted for 24 h without (fasted) or with refeeding for 6 h (refed) (n = 3, each). *F*, body weight curve from 4-week-old to 24-week-old of Armc5 flox (flox) or AdArmc5 KO (KO) (n = 9, each). *G*, wet weight of the indicated tissues of 24-week-old Armc5 flox (flox) or AdArmc5 KO (KO) fasted for 24 h followed by refeeding for 6 h (n = 3–9, each). *H*-*J*, gene expression of the indicated genes in the epididymal WAT (H), the liver (I), and the BAT (J) from 24-week-old Armc5 flox (flox) or AdArmc5 KO (KO) fasted for 24 h followed by refeeding for 6 h (n = 6, each). ∗*p* < 0.05; ∗∗*p* < 0.01. Open triangle, the Frt sites; Filled triangle, the LoxP sites; WAT, epididymal WAT; Sub, subcutaneous WAT; epi, epididymal WAT; mes, mesenteric WAT.

To explore the role of adipocyte *Armc5 in vivo*, we generated AdArmc5 KO. Armc5-floxed mice (Armc5 flox) were generated by crossing *Armc5* mutant mice and *CAG-FLPe* mice to excise *lacZ* and neomycin resistance gene (NeoR) cassettes. AdArmc5 KO were generated by crossing adipocyte-specific *Adipoq-Cre* mice and Armc5 flox (Fig. 2*A*). DNA of *Armc5* was excised in the WAT and the brown adipose tissue (BAT), but not in the liver of AdArmc5 KO (Fig. 2*A*). Gene expressions of *Armc5* were significantly decreased in the WAT and the BAT, but not in the liver of AdArmc5 KO, compared with those of Armc5 flox (Fig. 2*B*). Gene expression of *Armc5* was significantly blunted in the mature adipocyte fraction (MAF), but not in the stromal vascular fraction prepared from the epididymal WAT of AdArmc5 KO (Fig. 2*C*). Gene expressions of *Armc5* were also diminished in the MAF of mesenteric WAT and subcutaneous WAT from AdArmc5 KO (Fig. 2*D*).

### Downregulation of *Scd1* in the WAT of AdArmc5 KO

As we recently discovered that ARMC5 targeted full-length SREBF for ubiquitination and degradation (4), we first focused on SREBF pathway in AdArmc5 KO. Considering *Srebf1* was upregulated in the fasting-refeeding transition in the WAT of C57BL/6J mice (Fig. 2*E*), we analyzed AdArmc5 KO in the refed state. The body weight was not different between AdArmc5 KO and Armc5 flox until 24-week-old (Fig. 2*F*), and the weight of the WAT, the BAT, and the liver of AdArmc5 KO were similar to those of Armc5 flox in the refed state (Fig. 2*G*). Differently from our initial hypothesis that deletion of *Armc5* would increase SREBF protein and upregulate SREBF-target genes, gene expression of *Scd1*, one of SREBF1-target genes, was severely blunted, although other lipogenic genes (*Acaca*, *Fasn*, and *Acly*), cholesterol-related genes (*Ldlr* and *Hmgcr*), *Srebf1*, and *Srebf2* were not significantly changed in the epididymal WAT of AdArmc5 KO (Fig. 2*H*). Gene expression of these genes, including *Scd1*, were not changed in the liver (Fig. 2*I*) or the BAT (Fig. 2*J*) of AdArmc5 KO. The specific downregulation of *Scd1* was reminiscent of the phenotype of global *Srebf1* KO mice (13) or mice with adipocyte-specific overexpression of *Insig1* (14), where the levels of lipogenic genes were essentially unchanged except for reduction of *Scd1* in the WAT under normal chow.

### Impaired biosynthesis of unsaturated fatty acids pathway in global gene expression analysis

**Figure 3:**
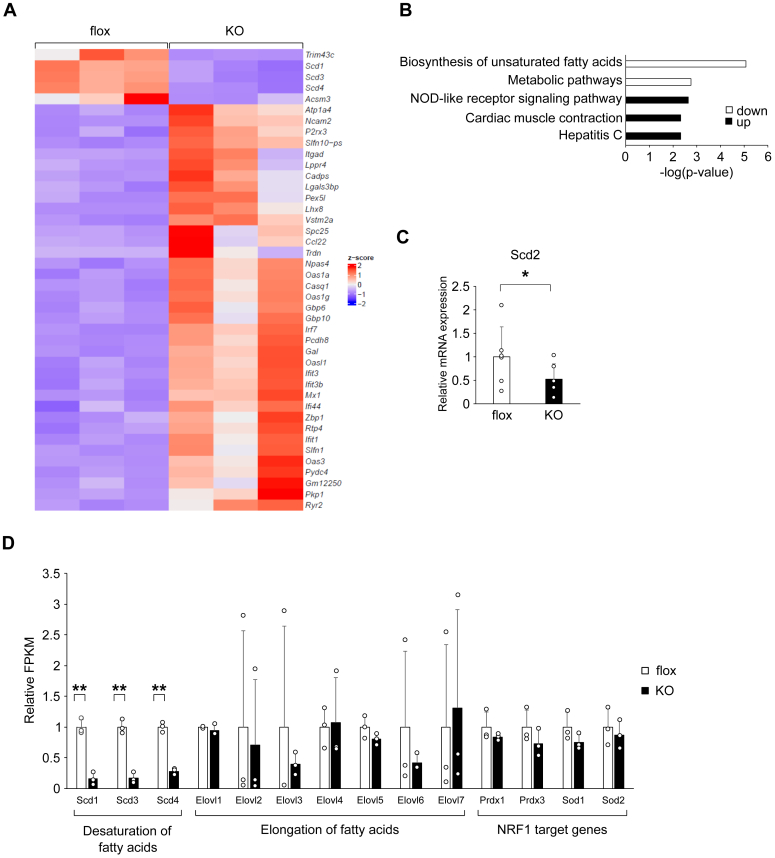
**Impaired biosynthesis of unsaturated fatty acid pathway in global gene expression analysis.***A*, RNA-Seq heatmap showing the most upregulated and downregulated genes (FDR <0.05 and fold change >3.0) between epididymal WAT of 24-week-old Armc5 flox (flox) and those of AdArmc5 KO (KO) fasted for 24 h followed by refeeding for 6 h (n = 3, each). *B*, ingenuity pathway analysis showing significantly altered canonical pathways of DEGs (FDR <0.1 and fold change >1.5) between epididymal WAT of 24-week-old Armc5 flox and those of AdArmc5 KO fasted for 24 h followed by refeeding for 6 h (n = 3, each). The pathways are indicated on the y-axis. On the x-axis, the significance score (negative log of *p*-value calculated by Fisher exact test) for each pathway is indicated by the bars. *White* bars and *black* bars indicate a prediction of an overall decrease and increase in the activity of the pathway in AdipoArmc5 KO, respectively. *C*, gene expression of Scd2 in the epididymal WAT of 24-week-old Armc5 flox (flox) or AdArmc5 KO (KO) fasted for 24 h followed by refeeding for 6 h quantified by RT-qPCR (n = 6, each). *D*, relative fragments per kilobase of exon per million mapped fragments (FPKM) in the RNAseq of the indicated genes in the epididymal WAT of 24-week-old Armc5 flox (flox) or AdArmc5 KO (KO) fasted for 24 h followed by refeeding for 6 h (n = 3, each). ∗*p* < 0.05; ∗∗*p* < 0.01.

To gain global and more insights about the roles of adipocyte *Armc5*, we next performed RNA-seq using epididymal WAT of Armc5 flox and AdArmc5 KO in the refed condition. *Scd1*, *Scd3*, and *Scd4* were among the five most downregulated genes that satisfied false discovery rate (FDR) < 0.05 and fold change >3.0 (Fig. 3, *A* and *D*). Consistently, the most downregulated pathway in AdArmc5 KO was ‘biosynthesis of unsaturated fatty acids’ among genes that satisfied FDR <0.1 and fold change >1.5 (Fig. 3*B*). *Scd2* was also downregulated in the epididymal WAT from AdArmc5 KO when evaluated by real-time quantitative PCR (RT-qPCR) in other set of animals (Fig. 3*C*). Although NRF1 was reported to be a substrate of ARMC5-CUL3 ubiquitin ligase (6), its target genes, *Prdx1*, *Prdx3*, *Sod1*, and *Sod2*, were not significantly changed in the WAT of AdArmc5 KO (Fig. 3*D*).

### Impaired fatty acid desaturation in the WAT of AdArmc5 KO

**Figure 4:**
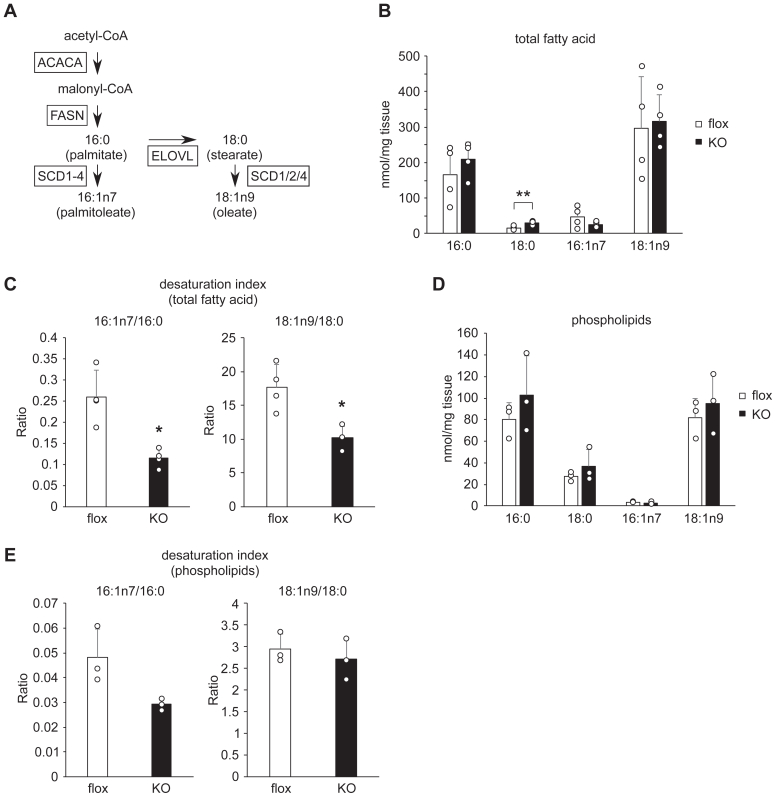
**Impaired fatty acid desaturation in the WAT of AdArmc5 KO.***A*, a scheme of the *de novo* lipogenesis pathway in rodents. *B* and *D*, fatty acid composition of total lipids (*B*) (n = 4, each) or phospholipids (*D*) (n = 3, each) of epididymal WAT from 24-week-old Armc5 flox (flox) or AdArmc5 KO (KO) fasted for 24 h followed by refeeding for 6 h. *C* and *E*, 16:1n7/16:0 and 18:1n9/18:9 desaturation indexes of total lipids (*C*) (n = 4, each) or phospholipids (*E*) (n = 3, each) of the epididymal WAT from 24-week-old Armc5 flox (flox) or AdArmc5 KO (KO) fasted for 24 h followed by refeeding for 6 h ∗*p* < 0.05; ∗∗*p* < 0.01.

Figure 4*A* illustrates the *de novo* lipogenesis pathway in rodents. 16:0 is synthesized from malonyl-CoA and is elongated by ELOVL and/or desaturated by SCD. Consistent with the downregulation of *Scd1-4* (Fig. 3, *A*–*D*) and unchanged expression of *Elovl1-7* (Fig. 3*D*), 18:0 significantly increased, 16:1n7 tended to decrease (*p* = 0.22) (Fig. 4*B*), and desaturation indexes (the ratio of 16:1n7 to 16:0 and 18:1n9 to 18:0) significantly decreased (Fig. 4*C*) in the epididymal WAT of AdArmc5 KO in the refed condition. In the WAT, fatty acids exist in the form of triglycerides (TG), cholesterol esters, and phospholipids. As fatty acid composition (Fig. 4*D*) and desaturation indexes (Fig. 4*E*) of phospholipids in the WAT were not different between genotypes, TG or cholesterol esters would be responsible for the impaired desaturation in the total fatty acids. From the data mentioned, *Armc5* was quite essential for the gene expression of *Scd* and fatty acid desaturation in the WAT under refed state.

### Downregulation of lipogenic genes in the WAT of AdArmc5 KO under high fat-high sucrose diet

**Figure 5:**
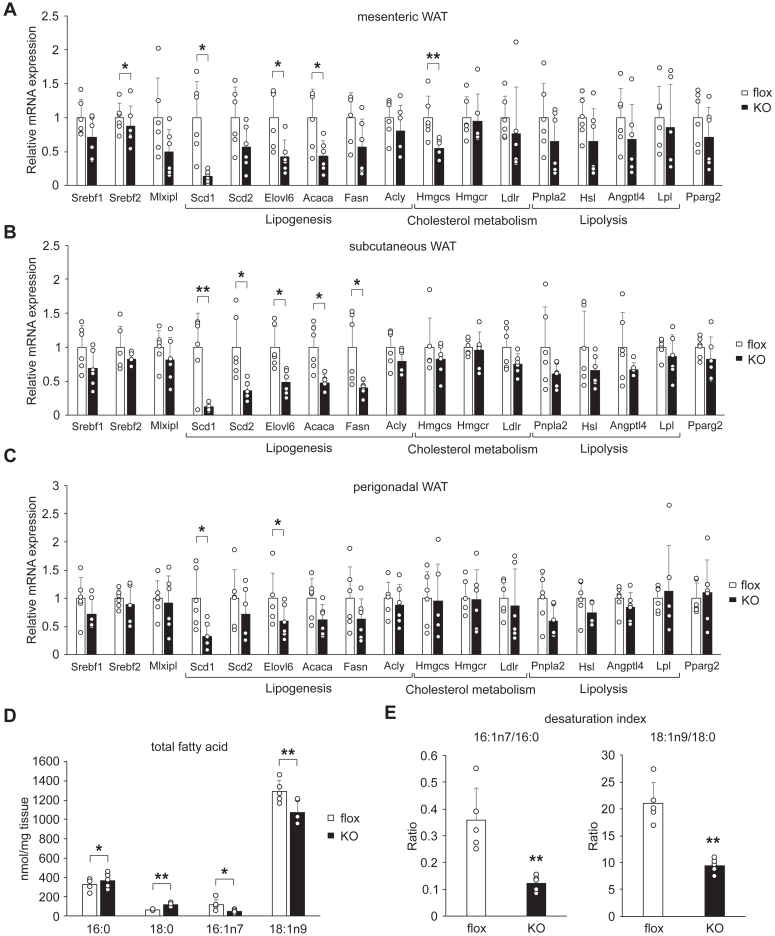
**Downregulation of lipogenic genes in the WAT of AdArmc5 KO under HF/HSD.***A*-*C*, gene expression of the indicated genes in the mesenteric WAT (A), the subcutaneous WAT (B), and the perigonadal WAT (C) of Armc5 flox (flox) or AdArmc5 KO (KO) fed a HF/HSD for 24 weeks (n = 6, each). *D*, fatty acid composition of the mesenteric WAT in Armc5 flox (flox) or AdArmc5 KO (KO) fed a HF/HSD for 24 weeks (n = 5, each). *E*, 16:1n7/16:0 and 18:1n9/18:9 desaturation indexes of the mesenteric WAT from Armc5 flox (flox) or AdArmc5 KO (KO) fed a HF/HSD for 24 weeks (n = 5, each). ∗*p* < 0.05; ∗∗*p* < 0.01.

Next, to explore the role of adipocyte *Armc5* in obesity, we fed AdArmc5 KO with a high fat-high sucrose diet (HF/HSD) from 4-week-old. The body weight was similar between genotypes until 28-week-old (Fig. S1*A*). Blood glucose, plasma insulin, glucose tolerance, and insulin sensitivity were also similar between genotypes (Fig. S1, *B*–*E*). Gene expressions were evaluated in each adipose depot (mesenteric, subcutaneous, and perigonadal) from AdArmc5 KO using RT-qPCR (Fig. 5, *A*–*C*). Similarly seen in the refed state, gene expression of *Scd1* was severely blunted in all the depots, but differently from the WAT in the refed state, several lipogenic genes (*Elovl6*, *Acaca* and *Fasn*) were significantly or tended to be decreased. Cholesterol metabolism–related genes were not changed except for the *Hmgcs* in the mesenteric WAT. Lipolysis-related genes (*Pnpla2*, *Hsl*, *Angptl4*, and *Lpl*), *Mlxipl*, and *Pparg2* were not changed. In the BAT of AdArmc5 KO, these genes were not changed except for slight increase of *Hmgcs* and *Pparg* (Fig. S2). The impaired fatty acid desaturation in the WAT was more apparent under HF/HSD compared with those in the refed state. 16:0 and 18:0 were significantly increased, 16:1n7 and 18:1n9 were significantly decreased (Fig. 5*D*), and desaturation indexes were significantly decreased (Fig. 5*E*). These data indicated that *Armc5* had a greater role in the regulation of fatty acid metabolism under diet-induced obesity compared with refed condition.

### ARMC5 regulated *Scd* gene expression through SREBF1 in a cell-autonomous manner

**Figure 6:**
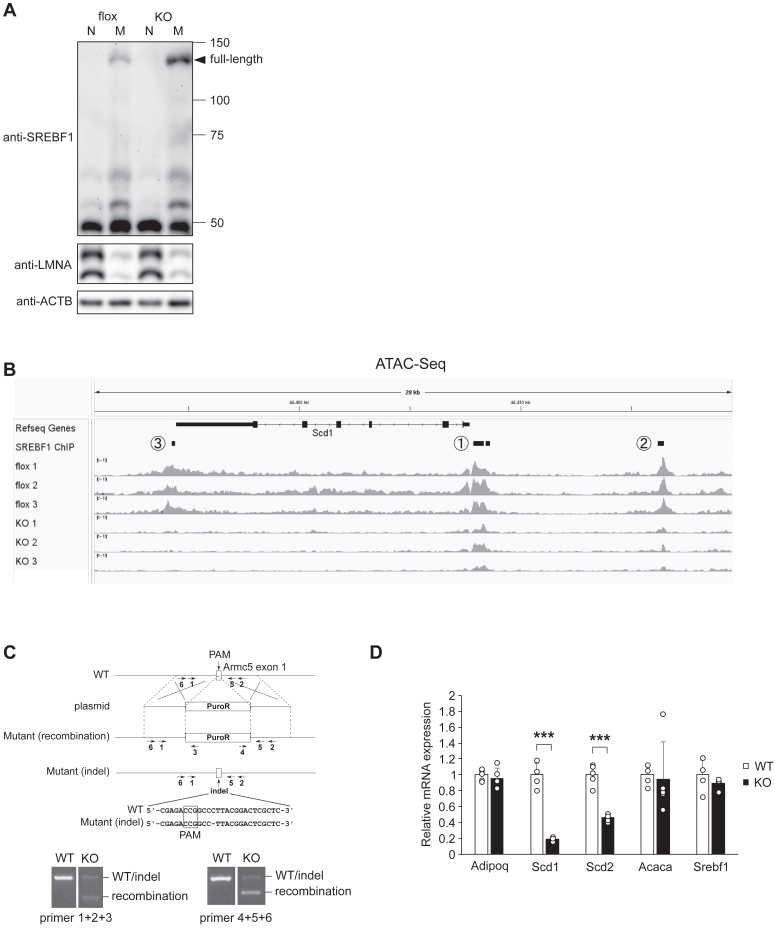
**ARMC5 regulated Scd gene expression through SREBF1 in a cell-autonomous manner.***A*, Western blotting with the indicated antibodies of the TCA/acetone precipitates of lysates from nuclear fraction (N) and membrane fraction (M) of the epididymal WAT of 24-week-old Armc5 flox (flox) or AdArmc5 KO (KO) fasted for 24 h followed by refeeding for 6 h (pooled sample from 4 mice). *B*, genomic tracks showing ATAC-Seq signals across the Scd1 locus for the epididymal WAT of 24-week-old Armc5 flox (flox) or AdArmc5 KO (KO) fasted for 24 h followed by refeeding for 6 h (n = 3, each). SREBF1-binding sites were collected from ChIP-Atlas (https://chip-atlas.org, accessed on 26 Dec 2023). Numbers with circle correspond to site 1, site 2, and site 3 in the manuscript. *C*, a strategic scheme (*upper*) and genotyping (*bottom*) for CRISPR/Cas9-mediated disruption of Armc5 in 3T3-L1 cells. Armc5 on one allele was disrupted by the insertion of PuroR (Mutant (recombination)) and Armc5 on the other allele was disrupted by the indel mutation (Mutant (indel)). Arrows with number represent the primers used in genotyping. The sequence shows the deletion in Mutant (indel). *D*, gene expressions of indicated genes in the parental 3T3-L1 adipocytes (WT) or 3T3L1-Armc5-KO (KO) 9 days after adipogenic induction (n = 5, each). ∗∗∗*p* < 0.001.

To investigate whether SREBF1 was involved in the downregulation of *Scd1*, we attempted to evaluate the amount of full-length SREBF1 and nuclear SREBF1 in the WAT of AdArmc5 KO. As shown in Fig. 6*A*, full-length SREBF1 was increased in the membrane fraction in the WAT of AdArmc5 KO. Unfortunately, we could not detect nuclear SREBF1 in the nuclear fraction probably due to technical difficulties of extracting sufficient nucleus from the WAT. So, we employed assay for transposase-accessible chromatin with high-throughput sequencing (ATAC-Seq), which is more sensitive than western blotting, in the WAT of AdArmc5 KO under refed condition. In the control flox mice, ATAC-Seq peaks well corresponded to three SREBF1-binding sites around the *Scd1* locus, located at the proximal promoter (site 1), 8.7 kb upstream of transcription start site (site 2) and near 3′ end of *Scd1* gene (site 3) (Fig. 6*B*). These ATAC-Seq peaks were drastically decreased in the WAT of AdArmc5 KO compared with those of Armc5 flox (Fig. 6*B*). These data suggested that loss of *Armc5* diminished SREBF1 activity due to impaired processing from full-length SREBF1 to nuclear SREBF1.

Next, to elucidate whether downregulation of *Scd* in the WAT of AdArmc5 KO was cell-autonomous, we disrupted *Armc5* in 3T3-L1 cells using CRISPR-Cas9 system. We obtained a clone (referred as 3T3L1-Armc5-KO) where biallelic *Armc5* was disrupted by the insertion of puromycin resistance gene (PuroR) in one allele and introduction of indel mutation in the other allele (Fig. 6*C*). The adipogenic capacity of 3T3L1-Armc5-KO was equivalent to parental 3T3-L1 cells as evidenced by similar *Adipoq* expression (Fig. 6*D*). Consistent with the findings in the WAT of AdArmc5 KO in the refed condition (Fig. 2*H*), gene expressions of *Scd1* and *Scd2*, but not *Acaca*, were severely blunted in the differentiated 3T3L1-Armc5-KO compared with the parental 3T3-L1 adipocytes (Fig. 6*D*).

### ARMC5 eliminated SCAP-free SREBF1

**Figure 7:**
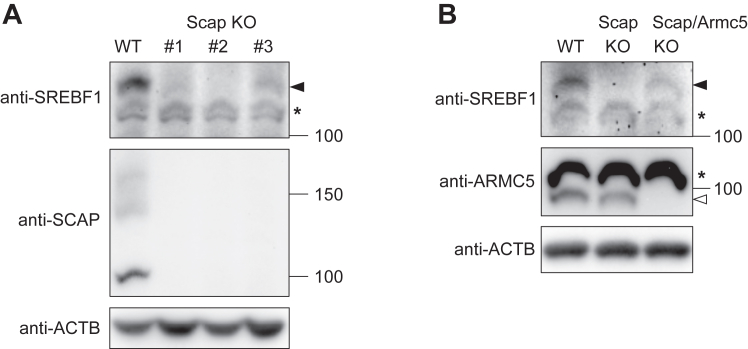
**ARMC5 eliminated SCAP-free SREBF1.***A*, Western blotting of lysates from the parental CHO-K1 cells (WT) or different clones of CHO-Scap-KO (Scap KO) with the indicated antibodies. *B*, Western blotting of lysates from the parental CHO-K1 cells (WT), clone #2 of CHO-Scap-KO (Scap KO), or CHO-Scap/Armc5 KO (Scap/Armc5 KO) with the indicated antibodies. Filled arrow, full-length SREBF1; open arrow, ARMC5; asterisk, nonspecific bands; arrow; full-length SREBF1.

The data so far indicated that ARMC5 was required for SREBF1 activity *in vivo*. However, it was counterintuitive because we recently reported that ARMC5 is an E3 ubiquitin ligase for full-length SREBF1 *in vitro* (4). To circumvent these issues, we focused on SCAP, an escort protein required for processing from full-length SREBF1 to nuclear SREBF1. To be noted, it was previously reported that full-length SREBF1 was actively degraded in the *Scap*-deficient CHO cells (15) or *Scap*-deficient liver (16), which predicted the unknown degradation machinery specific for SREBF1 that did not interact with SCAP (SCAP-free SREBF1). So, we speculated that ARMC5-CUL3 may mediate such degradation and generated Scap-deficient CHO cells by CRISPR-Cas9 system (referred as CHO-Scap-KO). As CHO-Scap-KO was supposed to be auxotrophic for cholesterol, mevalonate, and fatty acids (15), we supplemented these elements in the culture medium. We obtained three clones where *Scap* was disrupted by biallelic recombination of *PuroR* in the *Scap* gene (Fig. S3*A*). Consistently with the previous observations (15), full-length SREBF1 was severely decreased in all clones of CHO-Scap-KO (Fig. 7A). The band of full-length SREBF1 was verified using CHO cells introduced with siRNA targeting *Srebf1* (Fig. S4). In the CHO-Scap/Armc5 KO where *Armc5* was additionally disrupted by CRISPR-Cas9 system in CHO-Scap-KO (Fig. S3*B*), full-length SREBF1 was clearly restored at the level close to that in the WT CHO cells (Fig. 7*B*). These data indicated that ARMC5 was necessary for the removal of SCAP-free SREBF1.

### ARMC5 did not degrade SREBF1 and enhanced processing of SREBF1 in the presence of sufficient SCAP

**Figure 8:**
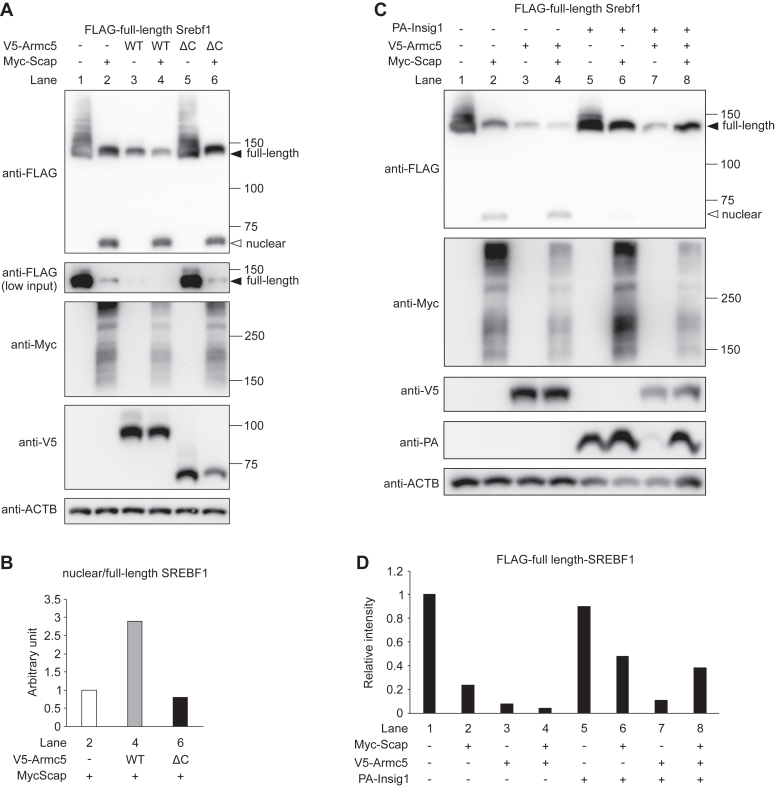
**ARMC5 selectively eliminated SCAP-free SREBF1 and enhanced processing of SREBF1.***A*, Western blotting of lysates with the indicated antibody from the HEK293T cells transfected with pcDNA3.1-FLAG-mSrebf1 and pRK5-HA-Ubiquitin-WT, together with pcDNA3.1-myc (−), pcDNA3.1-V5-Armc5 (WT), pcDNA3.1-V5-Armc5ΔC (ΔC), and/or pcDNA3.1/Hygro(+)-2xMyc-SCAP (Myc-Scap) for 24 h. 0.75% or 0.075% (low input) of lysate per lane was used for the detection of FLAG. *B*, relative band intensity of FLAG-nuclear SREBF1 to those of FLAG full-length SREBF1 in the lane 2, 4, 6 of (A).*C*, Western blotting of lysates with the indicated antibody from the HEK293T cells transfected with pcDNA3.1-FLAG-mSrebf1 and pRK5-HA-Ubiquitin-WT, together with pcDNA3.1-V5-Armc5 (V5-Armc5), pcDNA3.1/Hygro(+)-2xMyc-SCAP (Myc-Scap), and/or pcDNA3.1-PA-Insig1 (PA-Insig1) for 24 h. *D*, relative band intensity of FLAG full-length SREBF1 in (*C*). Filled arrow, FLAG-full-length SREBF1; open arrow, FLAG-nuclear SREBF1.

To elucidate whether ARMC5 degrades SREBF1/SCAP complex as well as SCAP-free SREBF1, *Srebf1*, *Scap*, and *Armc5* were cotransfected into HEK293T cells. Comparing lane 1 and 2 in Fig. 8*A*, overexpression of *Scap* expectedly promoted the processing of SREBF1 as was evident from decreased full-length SREBF1 and increased nuclear SREBF1 (two panels of anti-FLAG with different amounts of input are shown to precisely evaluate the protein level of full-length SREBF1). The band of nuclear SREBF1 was verified by nuclear fractionation (Fig. S5). In the absence of exogenous *Scap*, overexpression of *Armc5* markedly decreased full-length SREBF1 (Fig. 8*A*, lane 3 *versus* 1) in agreement with our previous report (4). In contrast, in the presence of exogenous *Scap*, overexpression of *Armc5* resulted in minor decrease of full-length SREBF1 (Fig. 8*A*, lane 4 *versus* 2), and the amount of nuclear SREBF1 was not changed, hence the ratio of nuclear SREBF1 to full-length SREBF1 was increased by the overexpression of *Armc5* (Fig. 8*A*, lane 4 *versus* 2 and Fig. 8*B*). Overexpression of Armc5ΔC, which lacks the BTB domain required for interaction with CUL3 (4), exerted essentially no effects on the amount and processing of SREBF1 with or without exogenous SCAP (Fig. 8, *A* and *B*).

Next, to rule out the potential influence of SCAP-mediated SREBF1 processing on the amount of full-length SREBF1, we suppressed SREBF1 processing by additional overexpression of *Insig1* that facilitates the retention of the SCAP/SREBF complex in the ER (17) (Fig. 8*C*). Expectedly, overexpression of *Insig1* significantly inhibited SCAP-mediated SREBF1 processing as evidenced by decreased nuclear SREBF1 and increased full-length SREBF1 (lane 6 *versus* 2, Fig. 8, *C* and *D*). Under overexpression of *Insig1*, ARMC5 greatly diminished the protein level of full-length SREBF1 in the absence of SCAP (lane 7 *versus* 5, Fig. 8, *C* and *D*) but exerted no effect on the amount of full-length SREBF1 in the presence of SCAP (lane 8 *versus* 6, Fig. 8, *C* and *D*). Taken together, these data have demonstrated that ARMC5 selectively eliminated SCAP-free SREBF1 and increased processing of SREBF1 in the presence of SCAP.

**Figure 9:**
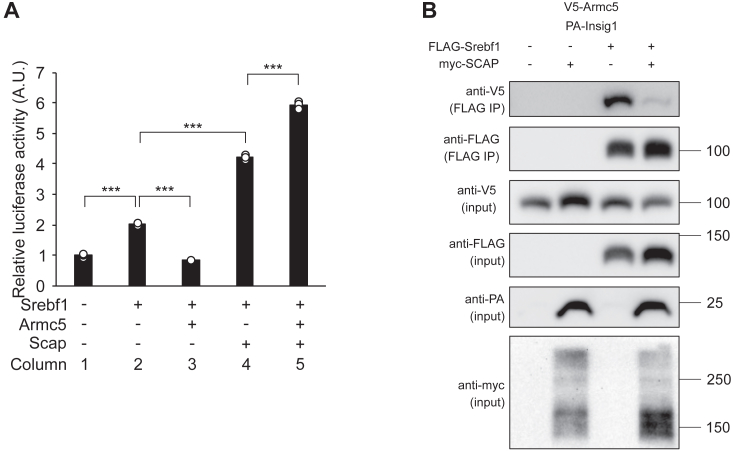
**The effect of SCAP on functional relationship between SREBF1 and ARMC5.***A*, pGL3-Scd1 reporter plasmid was transfected into HEK293T cells with pcDNA3.1-FLAG-mSrebf1 (Srebf1), pcDNA3.1-V5-mArmc5 (Armc5), and/or pcDNA3.1/Hygro(+)-2xMyc-SCAP (Scap). Normalized luciferase activities are shown (n = 3, each). ∗∗∗*p* < 0.001. *B*, Western blotting of lysates (input) or samples immunoprecipitated with anti-FLAG antibody (FLAG IP) from the HEK293T cells transfected with pcDNA3.1-FLAG-Srebf1 and/or pcDNA3.1/Hygro(+)-2xMyc-SCAP together with pcDNA3.1-FLAG-V5-Armc5 and pcDNA3.1-PA-Insig1 with the indicated antibodies.

This hypothesis was further validated by luciferase reporter assay (Fig. 9*A*) and co-immunoprecipitation assay (Fig. 9*B*). In HEK293T cells, the activity of *Scd1* promoter containing sterol response element (18) was activated by approximately two-fold by the overexpression of full-length *Srebf1* (column 2 *versus* 1, Fig. 9*A*), which was abrogated by cotransfection of *Armc5* (column 3 *versus* 2, Fig. 9*A*). In contrast, overexpression of *Scap* expectedly enhanced the activity of *Scd1* promoter (column 4 *versus* 2, Fig. 9*A*) and it was further activated by the overexpression of *Armc5* (column 5 *versus* 4, Fig. 9*A*). In the coimmunoprecipitation assay, HEK293T cells were transfected with full-length *Srebf1* and/or *Scap* together with *Armc5* and *Insig1*. We overexpressed *Insig1* because full-length SREBF1 would otherwise be processed by thw overexpression of *Scap* and we would not be able to properly evaluate the association between SREBF1 and ARMC5. As shown in Fig. 9*B*, overexpression of *Scap* severely blunted the physical interaction between ARMC5 and full-length SREBF1. These data were consistent with the hypothesis that SCAP is the deciding factor for ARMC5-mediated degradation of full-length SREBF1.

## Discussion

One of the major findings of the current study is that adipocyte *Armc5* was important for SREBF1 activity and fatty acid desaturation. There have been several evidence which support these findings. Firstly, in the systematic mapping of genetic interactions using genome-wide CRISPR library, *Armc5* was listed, alongside with *Ldlr*, *Fasn*, *Mbtps2* (*S2P*), *Scap*, and *Spring1*, as a gene which had negative genetic interaction with *Srebf2*, which indicated the involvement of *Armc5* in the SREBF1 pathway (19). Secondly, in the WAT of global *Srebf1* KO mice (13) or mice with adipocyte-specific overexpression of *Insig1* (14), the levels of lipogenic genes were essentially unchanged except for the reduction of *Scd1* under normal chow due to acute compensatory mechanism triggered by redox activation of mTORC1 and chronic compensation by ChREBP (14). In contrast, lipogenic genes were uniformly downregulated in the global *Chrebp* KO mice (20) or adipocyte-specific *Chrebp* KO mice (21). These literatures highlighted that, under the normal diet, SREBF1 upregulated a narrow subset of lipogenic genes, *Scd*, which was highly consistent with the gene profile in the WAT of AdArmc5 KO (Fig. 2*H*).

Another important finding is that ARMC5 selectively removed SCAP-free SREBF1 and accelerated the processing of SREBF1. Although we have already reported that ARMC5-CUL3 ubiquitinates and degrades full-length SREBF1 (4), it remained to be cleared whether ARMC5 targets SCAP-free SREBF1, SCAP-bound SREBF1, or both. In the current experiments, we uncovered that ARMC5-CUL3 selectively degraded SCAP-free SREBF1, but not SCAP-bound SREBF1. As the formation of SREBF1–SCAP complex was essential for transport from the ER to the Golgi, SCAP-free SREBF1 would not function properly and should be eliminated. In fact, previously, active removal of SCAP-free SREBF1 was strongly postulated (15, 16), but the underlying mechanisms and the physiological meaning of such removal, if any, had been unclear. From the experiments using CHO cells deficient for *Scap* and *Armc5* (Fig. 7), we have demonstrated that it should be ARMC5 that removes SCAP-free SREBF1. In contrast, ARMC5 did not degrade full-length SREBF1 in the presence of SCAP under condition where SREBF1 processing was constitutively suppressed by *Insig1* (Fig. 8, *C* and *D*). We further revealed that removal of SCAP-free SREBF1 enhanced the SCAP-mediated processing of SREBF1 (Fig. 8, *A* and *B*, Fig. 9*A*), which might explain the impaired SREBF1 activity in the WAT of AdArmc5 KO.

In Fig. 9*B*, we demonstrated that overexpression of *Scap* inhibited the physical interaction between ARMC5 and full-length SREBF1, but the mechanism is currently unknown. As ARMC5 interacts with the N-terminus of SREBF1 (4) and SCAP interacts with the C-terminus of SREBF1 (22), it seems unlikely that SCAP competitively shields the ARMC5-binding site of SREBF1. However, the relationships among SREBF1/ARMC5/SCAP were reminiscent of those of INSIG1/gp78/SCAP. Gp78 is the ubiquitin ligase of INSIG1 and physically interacts with and degrades INSIG1 in sterol-depleted cells. When SCAP binds to INSIG1 in sterol-loaded cells, SCAP liberates gp78 from INSIG1 through impaired physical interaction between gp78 and INSIG1, although the gp78-binding region and SCAP-binding region of INSIG1 seems different (23). So, we speculate that the binding of SCAP with SREBF1 causes steric hindrance towards ARMC5 similarly seen in INSIG1/gp78/SCAP.

**Figure 10:**
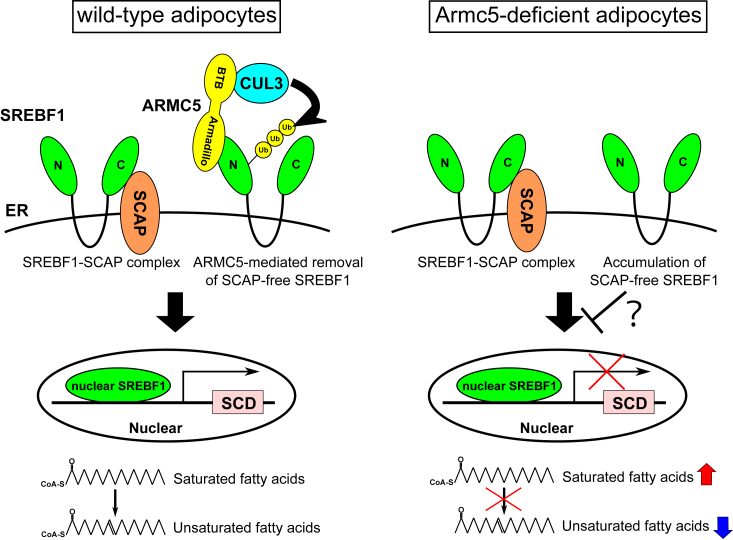
**Schematic representation of the possible roles of adipocyte Armc5 *in vivo*.** In the WT adipocytes, ARMC5–CUL3 complex selectively ubiquitinates and eliminates SCAP-free SREBF1. As a result, majority of SREBF1 form complex with SCAP and are appropriately processed to nuclear SREBF1, which upregulates Scd genes and enhances fatty acid desaturation (*left*). In the adipocytes deficient of Armc5, SCAP-free SREBF1 cannot be removed and are inadequately accumulated on the ER, which interfere the proper processing of SREBF1–SCAP complex. As a result, SREBF1 activity was diminished, leading to downregulation of Scd and impaired fatty acid desaturation (*right*).

In summary, we revealed that gene expression of *Scd* and fatty acid desaturation in adipocytes were highly dependent on ARMC5–SREBF1 axis *in vivo*. Mechanistically, we unveiled that ARMC5 selectively eliminated SCAP-free SREBF1and potentiated processing of SREBF1 (Fig. 10). ARMC5 may become a novel therapeutic target in the treatment of SREBF-related diseases, such as hepatosteatosis, dyslipidemia, atherosclerosis, and tumorigenesis (24).

## Experimental procedures

### Plasmids

Deletion mutants of *Srebf1* were generated by PCR from pcDNA3.1-FLAG-Srebf1(N) (4). pcDNA3.1/Hygro(+)-2xMyc-SCAP was a generous gift from Dr Sato (25). pcDNA3.1-FLAG-Srebf1, pcDNA3.1-myc-mArmc5, pcDNA3.1-FLAG-mArmc5, pRK5-HA-Ubiquitin-WT were described previously (4). pcDNA3.1-V5-Armc5 and pcDNA3.1-V5-Armc5ΔC was generated from pcDNA3.1-FLAG-mArmc5 and pRetroX-Tight-Pur-FLAG-mArmc5ΔBTB, respectively (4). The entire coding sequence of mouse *Insig1* was cloned and inserted into pcDNA3.1-PA to generate pcDNA3.1-PA-Insig1. Approximately 1 kb of mouse Scd1 promoter was cloned and inserted into pGL3-Basic (Promega) using the following primer. Forward; GCCTTTACCTTTGAGCCACTC and reverse; CTGAGATCGAGCGTGGACTTC.

### Cell culture

HEK293T cells and 3T3-L1 mouse fibroblasts (ATCC) were maintained in Dulbecco’s modified Eagle’s medium (high glucose) (Nacalai Tesque) containing 10% fetal bovine serum and penicillin/streptomycin (Nacalai Tesque). 3T3-L1 fibroblasts (ATCC) were differentiated into adipocytes by treatment with 2.5 μM dexamethasone (Nacalai Tesque), 2 μM insulin (Sigma Aldrich, I5500), 0.5 mM 3-isobutyl-1-methylxanthine (Nacalai Tesque), and 1 μM pioglitazone (Sigma-Aldrich, E6910) for 3 days, followed by 2 μM insulin for 3 days. CHO-K1 cells (ATCC) were maintained in D-MEM/Ham’s F-12 (Fujifilm WAKO) supplemented with 10% fetal bovine serum and penicillin/streptomycin (medium A). CHO-Scap-KO and CHO-Scap/Armc5 KO were maintained in medium A supplemented with 5 μg/ml cholesterol (Sigma-Aldrich), 1 mM mevalonic acid lithium salt (Sigma-Aldrich), and 20 μM sodium oleate (Sigma-Aldrich) (medium B).

### Transfection

Plasmids were transfected using lipofectamine 2000 (Thermo Fisher Scientific) according to the protocol provided by the manufacturer. Custom siRNA targeting to hamster *Srebf1* (siSrebf1) were purchased from Horizon Discovery. The sequence of siSrebf1 was 5′-GCACAGACCCAGUGGCCAAUU-3’. Twenty thousand of cells in 6-well plate was reverse-transfected with 10 pmol of siSrebf1 or siGENOME Control siRNA (Horizon Discovery) using Lipofectamine RNAiMAX Transfection Reagent (Thermo Fisher Scientific).

### Immunoprecipitation

Cells were lysed with TNE buffer [10 mM Tris–HCl, 150 mM NaCl, 1 mM EDTA, 1% NP40, and 1/100 Proteinase Inhibitor Cocktail (Nacalai Tesque)] and immunoprecipitated using anti-FLAG M2 Affinity Gel (Sigma-Aldrich), washed with TNE buffer, and eluted with 200 μg/ml FLAG peptide (Sigma-Aldrich).

### Western blotting

The cells or tissues were lysed with RIPA buffer [50 mM Tris–HCl (pH 7.8), 150 mM NaCl, 1 mM EDTA, 1% NP40, 0.5% sodium deoxycholate, 0.1% SDS, and 1/100 Proteinase Inhibitor Cocktail (Nacalai Tesque)] and subjected to Western blotting. ACTB was used for loading control. The blot signals were quantified using ChemiDoc Touch (Bio-Rad). For detection of SREBF1 in the WAT, lysates were concentrated by TCA/acetone precipitation. The antibodies used were anti-SCAP (Santa Cruz, sc-13553), anti-SREBF1 (Santa Cruz, sc-13551), anti-ACTB (Sigma-Aldrich, A5441), anti-CLAR (Cell Signaling, 12238), anti-LMNA (Cell Signaling, 2032), anti-ARMC5 (Novus Biologicals, NBP1-94024), anti-V5 (MBL, PM003), anti-HA (Cell Signaling, 3724), anti-PA (Fujifilm WAKO, 016-25861), anti-FLAG M2-HRP (Sigma-Aldrich, A8592), anti-Myc-HRP (Cell Signaling, 2040), HRP-linked anti-rabbit IgG (Cytiva, NA934), and HRP-linked anti-mouse IgG (Cytiva, NA931).

### *In vitro* ubiquitination assay

HEK293T cells were transfected with pcDNA3.1-FLAG-Srebf1 or pcDNA3.1-myc-mArmc5. Twenty-four hours after transfection, the cells were lysed with TNE buffer (10 mM Tris–HCl, 150 mM NaCl, 1 mM EDTA, 1% NP40, and 1/100 Proteinase Inhibitor Cocktail). The lysates were immunoprecipitated by anti-FLAG M2 affinity gel (Sigma-Aldrich) or anti-cMyc antibody beads (10D11) (Fujifilm Wako). The bound myc-ARMC5 was eluted by c-Myc peptide (Fujifilm Wako). The control sample was extracted from HEK293T cells transfected with empty plasmid and underwent the same purification steps. For *in vitro* ubiquitination reaction, immunoprecipitated FLAG-SREBF1 and purified myc-ARMC5 were added to a mixture containing 100 nM of UBE1 (R&D Systems), 1.4 μM of UbcH6 (BPS Bioscience), 50 μM of HA-Ubiquitin (R&D Systems), 500 nM of CUL3/RBX1 (R&D Systems), and 10 mM ATP in ubiquitination buffer (50 mM Hepes–KOH (pH 7.5), 50 mM NaCl, 5 mM MgCl, and 10 mM 2-mercaptoethanol). The reaction was carried out at 37 °C for 1 h. The beads were washed with TNE buffer, eluted with sample buffer (Fujifilm Wako), and subjected to western blotting.

### Animals

*Adipoq*-*Cre* mice were kindly provided by Dr Rosen (26). *Armc5* mutant mice, C57BL/6N-Armc5^tm1a(EUCOMM)Wtsi^/BayMmucd, were obtained from MMRRC (27). The *lacZ* and NeoR cassettes were removed from *Armc5* mutant mice by breeding with *CAG*-*FLPe* mice, C57BL/6-Tg(CAG-flpe)36Ito/ItoRbrc (RIKEN BRC) (28). The resultant Armc5 flox were crossed with *Adipoq*-*Cre* mice to generate AdArmc5 KO. The following primers were used to distinguish the null alleles from floxed allele. Primer 1, GCTTGATGGAATGCCAAGTTC; primer 2, TGCAATGACTTTGTGGGTCCATAAGC. In the experiments using Armc5 flox and AdArmc5 KO, the littermates were compared. Animals that have a bite wound were excluded. Randomization was not used. Animals were sacrificed alternately. The experimenters were aware of the group during sacrifice. In the experiments with a HF/HSD, the mice were fed F2HFHSD (Oriental Yeast) from 4 weeks of age. All mice were maintained under specific pathogen-free conditions and had free access to water and chow. Mice were anesthetized, blood was collected, and tissues were carefully removed and snap frozen in nitrogen. The experimental protocol was approved by the Ethics Review Committee for Animal Experimentation of Osaka University, Graduate School of Medicine. All animal experiments were carried out in accordance with the Institutional Animal Care and Use Committee Guidelines of Osaka University.

### mRNA analysis

Total RNA was isolated by TRI Reagent (Sigma-Aldrich) according to the protocol provided by the manufacturer. First-strand complementary DNA (cDNA) was synthesized from total RNA using the Transcriptor First Strand cDNA Synthesis Kit (Roche). cDNA was subjected to RT-qPCR using THUNDERBIRD SYBR qPCR Mix (TOYOBO) with LightCycler 96 System (Roche) according to the instructions provided by the manufacturer. The mRNA expression levels were measured relative to those of *Rplp0*. Relative mRNA expression is the value calculated relative to the standard samples in RT-qPCR. The primers used in this procedure are shown in Supplementary Table.

### Fractionation of the adipose tissue

MAF and stromal vascular fraction were isolated from each WAT depot of AdArmc5 KO or Armc5 flox fed normal chow as described previously (29).

### RNA-sequencing

Total RNA was extracted from tissues using TRI Reagent and RNeasy Mini kit (Qiagen) according to the manufacturer’s instructions. Library preparation was performed using a TruSeq stranded mRNA sample prep kit (Illumina) according to the manufacturer’s instructions. Whole transcriptome sequencing was applied to the RNA samples with the use of an Illumina NovaSeq 6000 platform in the 100-base paired-end mode. Sequenced reads were mapped to the mouse reference genome sequences (mm10) using TopHat ver. 2.0.13 in combination with Bowtie2 ver. 2.2.3 and SAMtools ver. 0.1.19. The number of FPKMs was calculated using Cuffnorm ver. 2.2.1. A heatmap was generated for DEGs with an FDR cutoff of 0.05 and a minimum fold change of 3 using RNAseqChef (https://imeg-ku.shinyapps.io/RNAseqChef/). iDEP.96 (http://bioinformatics.sdstate.edu/idep96/) was used for identification and enrichment analysis for DEGs with a FDR cutoff of 0.1 and minimum fold change of 1.5. Enrichment analysis for DEGs was performed using the gene sets of Kyoto Encyclopedia of Genes and Genomes.

### Fatty acid analysis

Total fatty acid saponification, extraction, and methylation were performed using commercial kits (Nacalai Tesque) according to the manufacturer’s protocol. Nonadecanoic acid (C19:0) was used as an internal standard. Fatty acid methyl esters were analyzed using gas chromatography–mass spectrometry (GC-MS) (QP2010, Shimadzu). The capillary column used for fatty acid separation was SP-2650 (100 m, inner diameter 0.25 mm, membrane thickness 0.20 μm, Sigma-Aldrich). The column temperature was maintained at 140 °C for 5 min, then increased gradually by 4 °C/min to 240 °C and held there for 20 min. The sample was injected in split mode with a split ratio of 1:25. Each fatty acid methyl ester was detected in the selected ion monitoring mode. All results were normalized to the peak height of the C19:0 internal standard and then quantified as nmol/mg tissue using Supelco 37 Component FAME Mix (Merck).

For fatty acid analysis of phospholipids, adipose tissues were homogenized using zirconia beads with 1 ml methanol containing 50 μl of 50 μM 1,2-dinonadecanoyl-sn-glycero-3-phosphocholine (19:0 PC; Merck KGaA) as an internal standard. Recovered supernatants were extracted by solid phase extraction on Isolute C18 columns (Biotage). The columns were washed with 6 ml of DW, 6 ml of hexane, and 6 ml of methyl formate. Phospholipids were eluted from the columns using 6 ml of methanol, dried under N2, and methylated with a commercially available kit (Nacalai Tesque) according to the manufacturer’s protocol. The concentrations of methylated fatty acids were measured using GC-MS (GC-MS QP2010 Plus, Shimadzu). The GC-MS conditions used for the measurements in this study were described in a previous study (30).

### Measurement of blood glucose and plasma insulin

Blood glucose was determined using the Glutest Sensor (Sanwa Kagaku Kenkyusho). Plasma insulin was measured using the insulin enzyme-linked immunoassay kit (Morinaga) according to the protocols supplied by the manufacturers.

### Glucose and insulin tolerance tests

Food was withheld for 4 h before glucose (1 g/kg) or insulin (1.3 units/kg for male and 1.1 units/kg for female) was administered intraperitoneally. Blood samples were collected from the tail vein at the indicated time intervals after injection. Blood glucose was immediately determined using the Glutest Sensor (Sanwa Kagaku Kenkyusho).

### Nuclear and ER membrane fractionation

The epididymal WAT was homogenized with a Dounce homogenizer (Active Motif) in ice cold homogenize buffer (20 mM Hepes (pH 7.4), 250 mM sucrose, 10 mM KCl, 2 mM MgCl2, 1 mM EDTA, and 1 mM EGTA and protease inhibitor cocktail). The nucleus was pelleted by centrifugation at 720*g* for 5 min. The pellet was resuspended in 10 ml of the same buffer and filtered through cheesecloth. The cleared sample was centrifuged again at 720 g for 10 min. The nuclear pellet was resuspended in the same buffer. The supernatant from the first 720 g spin was centrifuged at 16,500 g to recover ER-enriched membrane fractions. The ER pellet was resuspended in homogenize buffer. These fractions were subjected to Western blot analysis.

### ATAC-Seq

ATAC-Seq was applied to the epididymal WAT of Armc5 flox and AdArmc5 KO using the ATAC-Seq Kit (Active Motif) according to the manufacturer’s instructions. In brief, nuclei were isolated from the WAT and subjected to tagmentation reaction at 37 °C for 30 min using tagmentase-loaded (Diagenode). DNA was purified from the reaction mixture using DNA purification columns, and transposed fragments were amplified by 10 cycles of PCR using indexed primer pairs. The resulting libraries were sequenced using the Illumina NovaSeq 6000 platform in a 151-base paired-end mode. Adapter sequences were trimmed using Cutadapt v4.0. Trimmed reads were mapped to the mouse mm10 genome using Bowtie2 v2.3.5.1. The mapped reads were generated for visualization using DeepTools v3.5.1. The peaks were identified using MACS2 v2.2.7.1.

### Gene editing by CRISPR-Cas9

The CRISPR/Cas vector was based on pCCC kindly provided by Dr Lynn (31). To create 3T3L1-Armc5-KO, the gRNA (GAGAGCGAGTCCGTAAGGGC) was cloned into pCCC to generate pCCC-mArmc5. EF1 vector, which harbors *PuroR*, was generated from OCT4-eGFP-2A-Puro (Addgene, 31939). Homology arms were inserted into EF1 to generate EF1-mArmc5. The 3T3-L1 fibroblasts were transfected with pCCC-mArmc5 and EF1-mArmc5 and were subjected to limiting dilution. Genomic DNA from each clone was subjected to genotyping using the following primers. Primer 1, ATACCATTCTCGCCATGTCTTCTGC; primer 2, ATCTACTTCTGGTCTGGGCAGGTCC; primer 3, TTGGACAAACCACAACTAGAATGCAGTG; primer 4, TTGAATGGAAGGATTGGAGCTACGGGG; primer 5, AATCCCCTCTGCCGCCTTGATGTG; primer 6, AGATTCTCCCTGGTCACTTCTGGAGC. To create CHO-Scap-KO, the gRNA (TCTCACGCAGCCTTTCAGTC) was cloned into pCCC to generate pCCC-Scap. Homology arms were inserted into EF1 to generate EF1-Scap. The CHO-K1 cells were transfected with pCCC-Scap and EF1-Scap and were subjected to limiting dilution and maintained in medium B. Genomic DNA from each clone was subjected to genotyping using the following primers. Primer 1, AATTGCTTCTGGGGGTGTG; primer 2, ACTGCCACTTCGAGGAAAGAAAAAG; primer 3, CAAGGTTGTGCTTGTGCCTATATTG; primer 4, TTGCATTCCAGCTGACAGAC; primer 5, GCTTGGCTGGACGTAAACTC; primer 6, TCAGAAGCTGGTCGAGATCC. To create CHO-Scap/Armc5-KO, the gRNA (CAAGATGGCGGCTGCGAGAC) was cloned into pCCC to generate pCCC-cArmc5. *PuroR* of EF1 was replaced by hygromycin resistance gene (HygroR) to generate EF1-Hyg. Homology arms were inserted into EF1-Hyg to generate EF1-Hyg-cArmc5. CHO-Scap-KO were transfected with pCCC-cArmc5 and EF1-Hyg-cArmc5 and were subjected to limiting dilution and maintained in medium B. Genomic DNA from each clone was subjected to genotyping using the following primers. Primer 1, GGCTGTTTTGATGGTGAATG; primer 2, AAGGGAGAATGCCTCCAAGTC; primer 3, TTGAGCAGCACAGACTCCAC; primer 4, GCCAAATGAGCGAATACACAG; primer 5, GCCAGAGGCCACTTGTGTAG; primer 6, GTAGATGCCGACCGAACAAGAG.

### Luciferase reporter assay

0.25 μg of pGL3-Scd1 reporter plasmid, 0.25 μg of expression plasmids, and 2 ng of CMV-Renilla were transfected into HEK293T cells using lipofectamine 2000 (Thermo Fisher Scientific). The cells were harvested 24 h later. Luciferase assay was performed using Dual-Luciferase Reporter Assay System (Promega) according to the manufacturer’s instruction. Firefly luciferase activities were normalized by Renilla luciferase activities.

### Statistics

Data are presented as the mean ± SD. Differences between two groups were analyzed by 2-tailed *t* test using Excel. Significance was set at *p* < 0.05.

## Data availability

The raw data obtained in RNA-Seq in this study was submitted under Gene Expression Omnibus (GEO) accession number GEO DataSets: GSE262941. Any additional data presented in this paper are available from the corresponding author upon request.

## Conflict of interest

The authors declare that they have no conflicts of interests with the contents of this article.
