# Supplementary material for: ARMC5 selectively degrades SCAP-free SREBF1 and is essential for fatty acid desaturation in adipocytes
Source: J Biol Chem. 2024 Nov 2;300(12):107953. doi: 10.1016/j.jbc.2024.107953 (PMC11635738; doi:10.1016/j.jbc.2024.107953)
Supplement: Supplementary Figures [file mmc1.pdf]

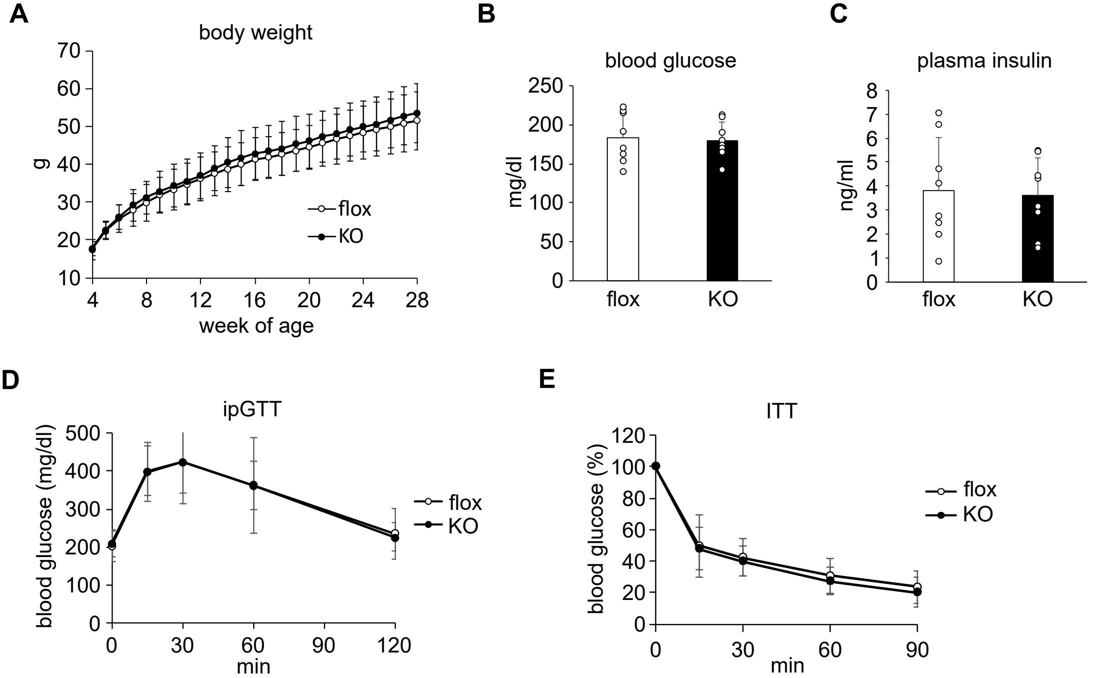

Supplementary Figure 1. Metabolic phenotype of AdArmc5 KO fed a HF/HSD. A: Body weight curve from 4 week old to 28 week old of Armc5 flox (flox) or AdArmc5 KO (KO) fed a HF/HSD (n=12, each). B, C: Blood glucose (B) and plasma insulin (C) of Armc5 flox (flox) or AdArmc5 KO (KO) fed a HF/HSD for 16 weeks (n=8, each). D: Intraperitoneal glucose tolerance test (ipGTT) of Armc5 flox (flox) or AdArmc5 KO (KO) fed a HF/HSD for 18 weeks (n=12, each). E: Insulin tolerance test (ITT) of Armc5 flox (flox) or AdArmc5 KO (KO) fed a HF/HSD for 21 weeks (n=11 , each).

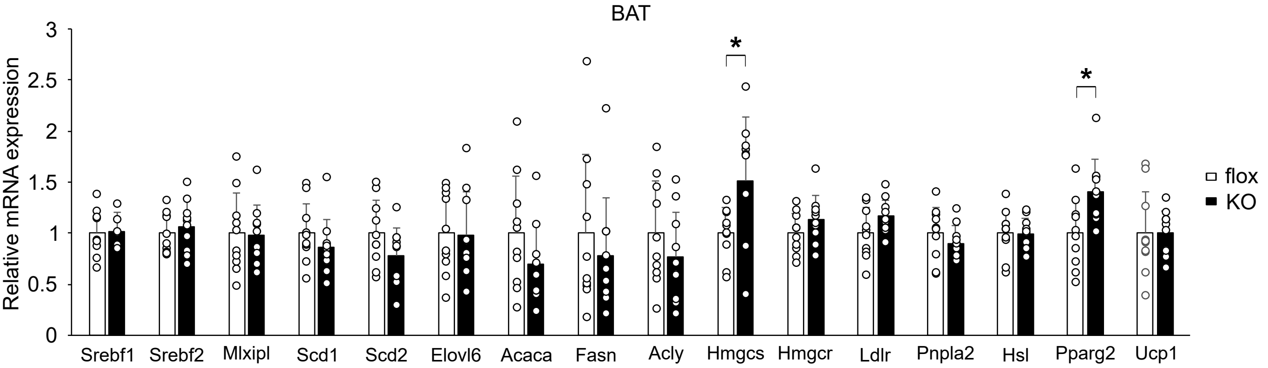

Supplementary Figure 2. Gene expression of the indicated genes in the BAT of Armc5 flox (flox) or AdArmc5 KO (KO) fed a HF/HSD for 24 weeks (n=9, each). *P<0.05.

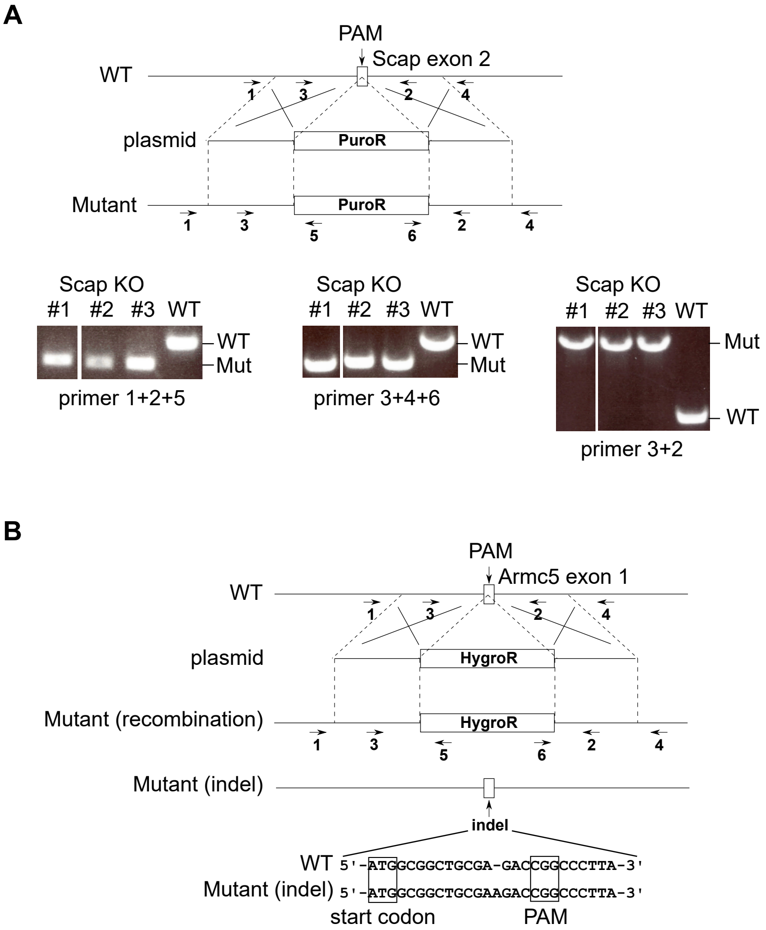

Supplementary Figure 3. A: Strategic scheme (upper) and genotyping (bottom) for CRISPR/Cas9-mediated disruption of Scap in the CHO-K1 cells. Arrows with number represent the primers used in genotyping. Scap KO; CHO-Scap-KO. WT; parental CHO-K1 cells. B: A strategic scheme for CRISPR/Cas9-mediated disruption of Armc5 in CHO-Scap-KO cells. Armc5 on one allele was disrupted by insertion of HygroR (Mutant (recombination)) and Armc5 on the other allele was disrupted by the indel mutation (Mutant (indel)). The sequence shows the insertion in Mutant (indel).

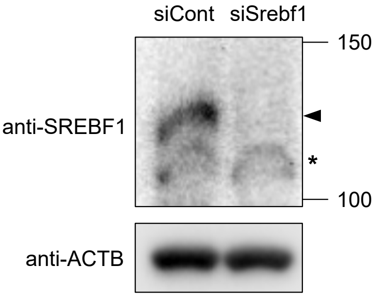

Supplementary Figure 4. Western blotting with the indicated antibodies of lysates from CHO-K1 cells introduced with siRNA targeting Srebf1 for 48 hours. Arrow; full-length SREBF1. Asterisk; non-specific band.

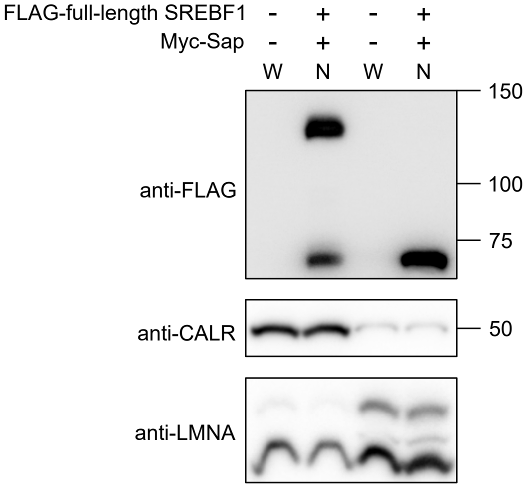

Supplementary Figure 5. Western blotting with the indicated antibodies of lysates of whole cell lysate (W) and nuclear fraction (N) from the HEK293T cells transfected with of without pcDNA3.1-FLAG-mSrebf1 and pcDNA3.1/Hygro(+)-2xMyc-SCAP for 24 hours.
