# Supplementary material for: ARMC5 selectively degrades SCAP-free SREBF1 and is essential for fatty acid desaturation in adipocytes
Source: J Biol Chem. 2024 Nov 2;300(12):107953. doi: 10.1016/j.jbc.2024.107953 (PMC11635738; doi:10.1016/j.jbc.2024.107953)
Supplement: Supplementary Tables [file mmc2.pdf]

Supplementary Table. The primers used in RT-qPCR.

	Forward primer	Reverse primer
mouse *Acaca*	AACTGGCCTTCTTGATGTTAGGAG	AGCACCGAGACTGAACTGTAAGG
mouse *Acly*	ACCCTTTCACTGGGGATCACA	GACAGGGATCAGGATTTCCTTG
mouse *Angptl4*	GCATGGCTGCCTGTGGTAAC	ATCTTGCTGTTTTGAGCCTTGA
mouse *Armc5*	TCCTGACCTGCACTTCGTTCTG	AGCGGCACCAAATCCATC
mouse *Elovl6*	GAAAAGCAGTTCAACGAGAACG	AGATGCCGACCACCAAAGATA
mouse *Fasn*	CATCCACTCAGGTTCAGGTG	AGGTATGCTCGCTTCTCTGC
mouse *Hmgcr*	GATTCTGGCAGTCAGTGGGAA	GTTGTAGCCGCCTATGCTCC
mouse *Hmgcs*	ACCACCAACGCCTGTTATGG	GCATAGCGACCATCCCAGT
mouse *Hsl*	CCGCTGACTTCCTGCAAGAG	CTGGGTCTATGGCGAATCGG
mouse *Ldlr*	GAGGAACTGGCGGCTGAA	GTGCTGGATGGGGAGGTCT
mouse *Lpl*	CCCTGAAGACACAGCTGAGG	GGCTGTACCCTAAGAGGTGG
mouse *Mlxipl*	CTCCGTAAGTCCAGCAGGGA	CGTCAGGTCTGGCTGGATCA
mouse *Pnpla2*	GGTGACCATCTGCCTTCCAG	TGCAGAAGAGACCCAGCAGT
mouse *Pparg2*	GCACTGCCTATGAGCACTTCAC	AGAGGTCCACAGAGCTGATTCC
mouse *Rplp0*	AAAGGAAGAGTCGGAGGAATCAG	GGCTGACTTGGTTGCTTTGG
mouse *Scap*	TGGAGCTTTTGAGACTCAGGA	TCGATTAAGCAGGTGAGGTCG
mouse *Scd1*	TGGGTTGGCTGCTTGTG	GCGTGGGCAGGATGAAG
mouse *Scd2*	GCATTTGGGAGCCTTGTACG	AGCCGTGCCTTGTATGTTCTG
mouse *Srebf1*	TGCTCCTGTGTGATCTACTTCTTG	TGGGGTCCATTGCTGGTAC
mouse *Srebf2*	CCCTTGACTTCCTTGCTGCA	GCGTGAGTGTGGGCGAATC
mouse *Adipoq*	GTTCTACTGCAACATTCCGG	TACACCTGGAGCCAGACTTG
hamster *Armc5*	TGCCTTGAAGCCTTTGTGCG	AGCAGCATCTCACCCAGCTC
hamster *Rplp0*	CTGCTGGCCAATAAGGTGCC	AGACCAGTGTTCTGGGCTGG
